# Biochar as Sustainable Alternative and Green Adsorbent for the Remediation of Noxious Pollutants: A Comprehensive Review

**DOI:** 10.3390/toxics11020117

**Published:** 2023-01-25

**Authors:** Stuti Jha, Rama Gaur, Syed Shahabuddin, Inderjeet Tyagi

**Affiliations:** 1 Department of Chemistry, School of Energy Technology, Pandit Deendayal Energy University, Knowledge Corridor, Raisan, Gandhinagar 382426, Gujarat, India; 2 Centre for DNA Taxonomy, Molecular Systematics Division, Zoological Survey of India, Ministry of Environment, Forests and Climate Change, Kolkata 700053, West Bengal, India

**Keywords:** biochar, adsorbent, wastewater treatment, heavy metals, dyes

## Abstract

The current water crisis necessitates the development of new materials for wastewater treatment. A variety of nanomaterials are continuously being investigated for their potential as adsorbents for environmental remediation. Researchers intend to develop a low-cost, simple, and sustainable material that can cater to removal of pollutants. Biochar derived from biowaste is a potential candidate for the existing problem of water pollution. The review focuses on the various aspects of biochar, such as its sources, preparation methods, mechanism, applications for wastewater treatment, and its regeneration. Compared with other adsorbents, biochar is considered as an environmentally friendly, sustainable, and cost-effective substitute for waste management, climate protection, soil improvement, wastewater treatment, etc. The special properties of biochar such as porosity, surface area, surface charge, and functional groups can be easily modified by various chemical methods, resulting in improved adsorption properties. Therefore, in view of the increasing environmental pollution and the problems encountered by researchers in treating pollutants, biochar is of great importance. This review also highlights the challenges and prospective areas that can be explored and studied in more detail in the future.

## 1. Introduction

Water pollution is increasing due to the different factors such as population growth, urbanization, industrialization, etc., which is a major concern worldwide. Environmental pollutants can cause various diseases, neurological problems, and cancer. The wastes generated by industries are released into the water bodies, atmosphere, and soil, contributing to the pollution of soil, water, and air. Pollution results in adverse effects on the entire ecosystem, especially on human life. Most pollutants that pose a threat to the environment originate from industry. Industrial wastewater, which consists of organic and inorganic substances, is toxic to humans and must therefore be treated before it can be released into the environment.

Researchers are continuously striving to reduce pollutants. Generating sustainable and green energy sources to meet the needs of today’s world is one such initiative. In recent years, researchers have also made efforts to find a sustainable, environmentally friendly solution to the pressing problem of waste treatment. Methods such as advanced oxidation processes, adsorption, ion exchange, reverse osmosis, ozonation, interfacial solar evaporation, coagulation, and precipitation are used to treat various pollutants [1,2,3,4,5,6,7,8,9]. All reported methods (except adsorption) are either associated with high operating costs or are inherently complex; therefore, these methods are not economically viable [10]. Adsorption is the most widely used technique because it is efficient, requires low investment, and is easy to set up [11], which raises the need to develop a cost-effective and potential adsorbent material for environmental remediation. There are several reports on the use of materials such as various nanomaterials, silica gel, activated alumina, hydrogels, zeolites, etc., for the adsorption process [12,13,14]. The search for low-cost and sustainable adsorbents with high adsorption capacity continues. Among the reported absorbents, the use of biowaste and biochar is a potential option and has great prospects.

The term “biochar” was first coined by Peter Read in 2015 [15]. Biochar is considered a promising candidate because it is a sustainable, cost-effective, and biowaste-based adsorbent. It is a pyrolytic or anaerobic, thermally treated, stable, and carbon-rich substance obtained from organic material as raw material [16]. Waste materials containing lignin or non-lignin from agriculture, aquaculture, wood, and fibre processing industries, etc., are primarily used for the production of biochar. Specific properties of biochar include hydrophobicity, long-term stability, chemical composition, large surface area, high porosity, etc. This makes it a potential candidate for multiple applications such as harmful pollutant removal, carbon sequestration, greenhouse gas adsorption, etc.

In addition, the surface properties of biochar can also be adjusted by various modification processes such as acid/alkali treatment, application of other materials to the surface, ball milling, acetylation, etc. [16,17,18]. From the literature, it is found that the processed or modified biochar has higher efficiency in removing pollutants compared with the original biochar [19]. Moreover, biochar has intrigued researchers as it provides a more environmentally friendly alternative for environmental management in soil improvement, waste management, energy production, and climate change mitigation [19]. Given the wide acceptance and recent advances in the field of biochar, this review focuses on (1) sources, properties, and various synthetic routes of biochar; (2) potential of biochar for remediation of harmful pollutants such as inorganic and organic contaminants and their adsorption mechanism; (3) correlation with the Sustainable Development Goals (SDGs), especially SDG 6; (4) and future prospects and recommendations, which highlight certain grey areas of biochar that remain to be explored in the future.

## 2. Sources of Biochar

**Figure 1:**
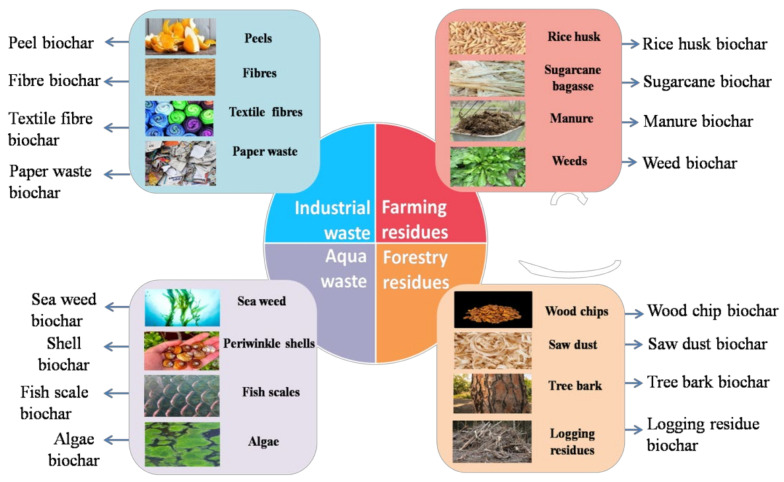
Different sources of raw materials and types of biochar.

The preparation of biochar does not involve use of harmful chemicals. It can be prepared simply by using any bio-waste as a raw material. Thus, as biochar is derived from green sources, it can be considered as a green adsorbent. In recent years, biochar has attracted much attention, mainly because of its application and the wide availability of raw materials that can serve as raw material. The raw materials for biochar are not only widely available but can also be easily collected. Any material that is rich in carbon can serve as a feedstock for biochar production. It can be obtained from agricultural and forestry waste, food, and fibre, processing industry waste, grassland, etc., as shown in Figure 1.

Based on the composition of biomass, it can be divided into lignocellulosic (with lignin and cellulose) and non-lignocellulosic (without lignin and cellulose) groups [20]. The content of inorganic components, nutrients, minerals, lignin, cellulose, moisture, hemicellulose, volatiles, carbon and ash fractions, density, and particle size of biomass determine the properties and performance of biochar [21,22]. The lignocellulosic group includes agricultural residues, forestry wastes, bioenergy crops, etc. The non-lignocellulosic group includes sewage sludge, manure, animal hair, algae, etc. [23]. As reported by Filiberto et al., non-lignocellulosic biomass has high mineral and nutrient composition compared with lignocellulosic biomass [24]. The various sources of raw materials for biochar are discussed in detail according to their origin as follows:

### 2.1. Farming Residues

**Figure 2:**
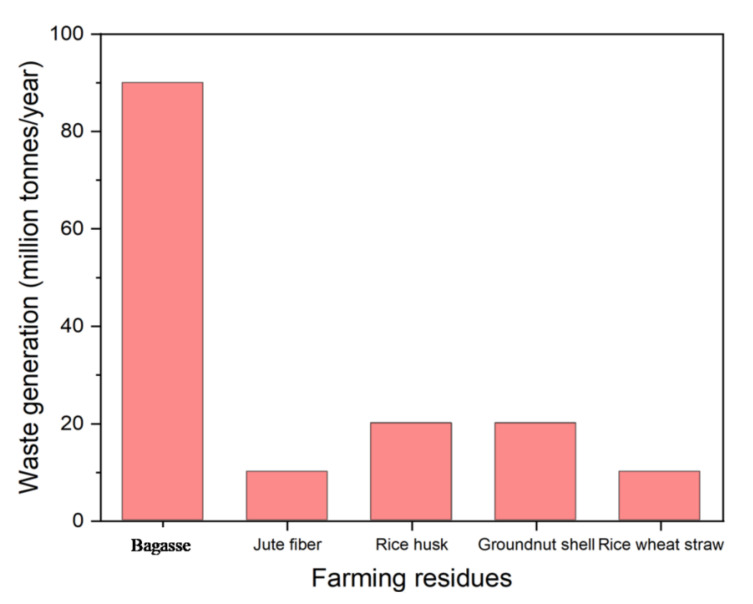
Graphical representation of the various agricultural wastes generated in India.

Agricultural waste is defined as unwanted material generated by various agricultural activities. These include cereal straw, bagasse, and fibres as shown in Figure 2 [25,26,27]. Compared with other countries in the world, India, whose economy is based on agriculture, is also one of the largest producers of agricultural waste in the world. According to a survey conducted in 2017, India produces an average of 516 million tons of crop residues each year [28]. Every year, the production of agricultural waste increases by about 7.5% [29]. The agricultural sector is considered to be one of the largest sources of agricultural solid waste generation, the disposal of which is a major problem for countries around the world. Improper disposal and management of agricultural waste such as weeds, crop residues, leaves, etc., have negative impacts on the environment.

Agricultural waste is considered the most important source for the production of biochar [23]. The composition of agricultural waste consists of cellulose (35–50%), lignin (15–25%), and hemicelluloses (15–40%) [30], which affect the properties of biochar produced. For example, rice husk was used by Nazia Hossain et al. [31] to synthesize biochar at a temperature of 180 °C and a pressure of 70 bar. Gopal et al. synthesized biochar using coconut shells, coconut leaves, and also coconut fibres [32]. On the other hand, Sarfaraz et al. developed biochar using animal dung as a low-cost raw material, they used dung of pig, cattle, poultry litter, etc. Moreover, they also used soybean straw and rice straw for the synthesis of biochar [33].

### 2.2. Forestry Residues

Forestry wastes are a rich source of lignocellulosic biomass [34]. Like agricultural waste, forestry waste is a second-generation feedstock used for the synthesis of biochar. According to a report by Food and Agriculture Organization (FAO), India consumes nearly 10,000 tons of wood, indicating the wide availability of forest wastes [35]. Wood French fries, tree bark, wood shavings, sawdust, etc., are among the forestry wastes that serve as feedstock for biochar. Volatile matter, lignin, pectin, and resin are present in biochar obtained from wood, which is why it has a high calorific value [34].

Although forestry residues are a potential source of biochar, they must be used with caution because they promote deforestation. Generally, pre-treatment of the forestry feedstock (chipping, crushing, and grinding) is required before it can be used for biochar production [36]. Lai et al. and Kanouo et al. reported the use of pyrolyzed wood French fries from cedar and eucalyptus trees to produce biochar [37,38].

### 2.3. Aquatic Residues

Aquatic biomass is rich in proteins, carbohydrates, and lipids, but has low lignin content compared with forest and agricultural residues [39]. Lignin-rich biomass such as forestry and agricultural residues require extensive treatment to break down their complex cell wall structure and are averse to further processing (chemical reactions) [40]. This results in the need to develop and use starch- or cellulose-rich biomass for biochar production. Aquatic biomass can be easily processed compared with other lignocellulosic biomass due to its simpler structure. Algae, kelp, fish scales, periwinkle shells, etc., which are part of aquatic biomass, are suitable raw materials for biochar production. Algae have high nutrient concentration and are an important feedstock for biochar production, but its applicability is limited due to high collection cost [41].

Cai et al. used biochar from crab shells for adsorption of diesel oil [42]. Roberts et al. used algae as feedstock to produce biochar by pyrolysis to improve soil quality [43]. The adsorption of copper ions on biochar synthesized from fish scales was reported by Achieng et al. [44].

### 2.4. Industrial Residues

Rapid industrialization has led to environmental degradation through industrial pollution. Wastes and by-products generated by various industries such as textiles, food, and paper are rich sources of carbon. Improper disposal and management of these wastes is one of the biggest problems in the world. Waste from industry causes air, water, and soil pollution, and emits harmful gases such as methane as it rots. Therefore, it is essential to ensure that industrial wastewater does not contribute to pollution. Converting industrial biomass into biochar contributes to both environmental protection and waste management.

The food, pulp, and paper industries are considered to be the industries with the highest waste generation [45]. Solid wastes from food processing industries such as pulp, fibres, peels, food, and vegetable wastes, etc., are used as raw materials for the production of biochar. Creamer et al. demonstrated the use of sugarcane bagasse as a source of biochar for CO_2_ adsorption [46]. Wu et al. studied the adsorption of dyes by biochar from litchi peels [47]. Textile fibres, which are waste from the textile industry, are also a potential biochar source for biochar production. Hanoglu et al. produced biochar from different types of textile fibres to investigate the properties of biochar [48]. Solid wastes from the pulp and paper industry mainly include sewage sludge, paper, wood residues, etc., which serve as biomass. Waste paper has high cellulose content, making it a suitable raw material for biochar. Xu et al. reported the production of biochar from waste art paper and the adsorption of lead ions [49].

## 3. Synthetic Routes

Due to the wide range of applications of biochar, the conversion of biomass into biochar has increased. To produce biochar, the raw material is collected and subjected to thermal decomposition under various conditions. Different processes are used to convert biomass (raw material) into biochar depending on the temperature range, conditions, and residence time, namely pyrolysis, hydrothermal carbonization, torrefaction, and gasification. All processes produce biochar (solid) as the main product, as well as bio-oil (liquid) and biogas (gaseous) in larger or smaller quantities. The percentage of solid, liquid, and gaseous products varies from method to method. The percentage of final products and the advantages and disadvantages of each method are summarized in Table 1. The synthetic method is selected depending on the biomass in order to obtain the maximum yield of biochar [50]. The properties of the feedstock and the method used for biochar production have a great influence on the physical and chemical properties of biochar, so it is crucial to understand the heating rate, residence time, and decomposition temperature of the different production methods. The methods used to produce biochar are explained in detail below:

### 3.1. Pyrolysis

Exposing the feedstock to a high temperature (250–900 °C) in the presence of limited or no oxygen is called pyrolysis. Pyrolysis has been reported to be the preferred method among all biochar production processes because it can be used for a wide range of biomass [51]. During pyrolysis, the components present in biomass such as cellulose, hemicellulose, and lignin are depolymerized, fragmented, and cross linked. This leads to the formation of products in different phases such as solid, liquid, and gas. Pyrolysis conditions such as temperature, residence time, and type of biomass play an important role in biochar yield [52]. Pyrolysis can be divided into slow pyrolysis, fast/flash pyrolysis, and microwave pyrolysis depending on the temperature and residence time.

#### 3.1.1. Slow Pyrolysis

In this process, the biomass undergoes pyrolysis at 300–500 °C with a longer residence time of several minutes to days [53]. Slow pyrolysis is considered the most effective because it yields a maximum of solid products (biochar) and a lower level of gaseous (biogas) and liquid products (bio-oil). In general, as the temperature increases, the yield of biochar decreases and the amount of biogas decreases. Since slow pyrolysis involves a slower heating rate and longer residence time, maximum biochar yield is obtained [54]. According to one study, slow pyrolysis achieves a biochar yield of 35.0% from dry biomass [55]. This is the main reason why slow pyrolysis is the most suitable method for biochar production among all pyrolysis processes.

#### 3.1.2. Fast Pyrolysis

Fast pyrolysis is a thermochemical process for liquefying biomass into bio-oil, which is highly useful for energy production [50]. It is carried out at a temperature above 500 °C to 700 °C. This type of pyrolysis involves faster heating rates (>10–10,000 °C/min) and shorter residence times in seconds. As mentioned earlier, the yield of biochar from this process is comparatively low compared with slow pyrolysis due to the increase in temperature and reduction in residence time. Instead, a maximum amount of bio-oil is obtained at the end of the fast pyrolysis. The type of pyrolysis process also affects the properties of biochar produced. A higher pyrolysis temperature results in biochar with a larger surface area, higher pH and volatiles, but lower surface functional group and cation exchange capacity (CEC) [55]. Ghani et al. demonstrated that biochar becomes more hydrophilic at temperatures below 500 °C (slow pyrolysis), while biochar exhibits hydrophobic behaviour and high thermal stability at temperatures above 650 °C [56].

#### 3.1.3. Microwave Pyrolysis

Microwave pyrolysis is the decomposition of biomass by energetic microwave radiation [19]. Unlike conventional pyrolysis, in microwave pyrolysis the temperature at the centre of the feedstock is higher than at the surface [57]. This results in efficient heat transfer and shorter reaction time compared with other thermochemical methods [58]. The process is cost effective as microwave pyrolysis provides rapid and uniform heating, i.e., energy intensive and steps such as preheating and dehumidification is not essentially required in this process [59,60]. However, microwave pyrolysis yields a very small amount of bio-oil compared with other conventional processes. Wang et al. produced biochar by microwave-assisted pyrolysis from camellia (Camellia oleifera) peel with a yield of 37.45% and 27.45% for biochar and bio-oil, respectively [61].

### 3.2. Hydrothermal Carbonization (HTC)

In hydrothermal carbonization, also known as “wet pyrolysis”, the biomass is surrounded by water during the reaction [62]. In this process, the reaction temperature is applied in a range of 180–230 °C under low pressure in a closed system [17]. In the HTC method, the pressure and temperature are maintained in such a way that the water remains in a liquid state throughout the process. Further, the advantages of HTC includes energy efficient production of biochar in high yield, i.e., low temperature requirement (about 180 °C) [31]. It involves a series of reactions such as hydrolysis, condensation, decarboxylation, and dehydration [63]. The HTC method for the production of biochar is attracting more and more attention because it offers the advantage of using wet biomass and does not require an additional drying step. To distinguish the biochar obtained by pyrolysis and HTC, the final product of HTC is called “hydrochar”. The hydrochar formed has more oxygen-containing functional groups, which makes it more favourable for adsorption of heavy metals from aqueous media [1]. Reports indicate that hydrochar has more oxygen-containing groups and higher CEC value than the biochar produced by pyrolysis, which is an important factor for the use of biochar as an adsorbent [64]. The easy biodegradability of hydrochar limits its application for carbon sequestration [65].

### 3.3. Torrefaction

Torrefaction is one of the new techniques for biochar production from biomass. In this process, raw material was heated for the residence time of 15–60 min within the temperature range 200–300 °C in an inert atmosphere [19]. Since, the operating temperature is lower than that of pyrolysis, it is also called “mild” pyrolysis [50]. In this process, biomass depolymerization occurs, the extent of which depends on the reaction time and temperature. Biochar is not the main product of torrefaction, but is suitable for adsorption of pollutants [53]. The biochar produced by this process exhibits hydrophobic properties because the hydroxyl groups present on the surface of the biochar are removed during this process [66]. In addition, the biochar obtained from torrefaction has better ignitability, grindability, high calorific value, carbon content, energy yield, and lower moisture content, which makes it more suitable for use as a fuel [67,68,69]. According to a study by Pathomrotsakun et al., biochar produced from coffee residues has a calorific value and energy yield of 31.12 MJ/kg and 48.04%, respectively [67].

### 3.4. Gasification

Gasification of biomass is shown as another alternative method for the synthesis of biochar. The biochar was synthesized in an oxidizing environment (single or mixture of gases) at a temperature of ~700° [41]. Further, the partial combustion of the feedstock under oxidizing atmosphere produces syngas, a mixture of H_2_, CO, CO_2_, and CH_4_, which is used as fuel. The reaction temperature is the key factor affecting the production of syngas [50]. Gasification produces the highest percentage of gaseous product and very little biochar compared with the other processes. The biochar obtained by this process is more stable and resistant to chemical oxidation, and has a smaller particle size. Biochar contains minerals such as N, K, P, and Ca which increases the soil fertility and enhances the growth of plants. The amount of biochar can be varied from 2590 kg ha^−1^ to 34.2 t ha^−1^ in studies reporting the use of biochar as a fertilizer as reported by Yang et al. and Oh et al., respectively [70,71,72].

Converting biomass into biochar is an effective strategy for waste management and environmental protection. Decomposition of organic materials releases harmful gases such as carbon dioxide and methane into the atmosphere, which contributes to global warming [73]. Through biochar production, solid waste issues of biomass can be managed and it can also prevent the production of greenhouse gases, thereby contributing towards the reduction in GHGs. Moreover, it reduces the cost associated with the solid waste disposal.

**Table 1:** Percentage yield of solid, liquid, and gaseous products obtained from biochar by various routes [74,75,76,77,78].

Method	Temperature (°C)	Conditions	Percent Yield of Products			Advantages	Disadvantages
			Solid (Biochar)	Liquid (Bio-Oil)	Gaseous (Biogas)		
Slow pyrolysis	300–500	Oxygen-free atmosphere	35	30	35	Highest yield of biochar	Further treatment of gases is needed
Fast pyrolysis	500–700	Oxygen-free atmosphere	12	75	13	Higher yield of bio-oil	Low biochar yield Fine particle biomass is required
Microwave pyrolysis	-	-	-	-	-	No size reduction or drying of biomass is required Rapid and uniform heating	Energy requirement is high
HTC	<230	Low pressure	50–80	5–20	2–5	Low operating temperature and residence time	Separation of solid and liquid phase
Torrefaction	200–300	Inert atmosphere	60	20	20	Zero waste process Upgraded quality of biochar	Feedstock sensitivity High investment cost
Gasification	>700	Oxidizing atmosphere	10	5	85	Reduced emissions High energy efficiency	Complex technology and high operation cost

## 4. Properties of Biochar

**Figure 3:**
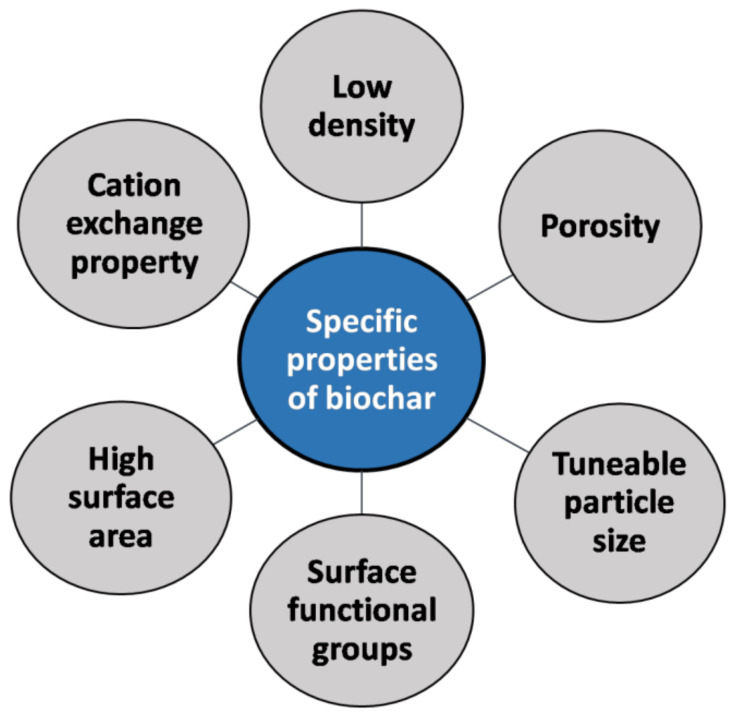
Specific properties of biochar.

The excellent performance of biochar as an adsorbent can be attributed to its characteristic properties. Figure 3 depicts the specific properties of biochar, i.e., porous nature, high carbon content, high specific surface area (SSA), hydrophobic nature, and ease of functionalization. Further, the composition of biochar in terms of carbon, nitrogen, sulfur, and oxygen affects the chemical properties of biochar [19]. The properties of biochar depend on several factors such as composition of the raw material, preparation method, and pyrolysis temperature. Thus, it is important to understand how the reaction conditions influence the property of biochar. Therefore, in order to develop biochar with desired properties or to treat the analyte of interest, it is inevitable to have a thorough knowledge of all the factors affecting its properties. In their study, Evans et al. demonstrated the effects of the feedstock on the properties of biochar. They produced biochar from various agricultural by-products (e.g., hardwood French fries, pine French fries, cotton litter, poultry litter, miscanthus grass, and switchgrass) as feedstocks under reaction conditions of 400 °C for 2 h. From this research, it was found that the mineral content of biochar varies greatly depending on the feedstock used [79]. Poultry litter biochar had a higher mineral content than all other types of biochar produced in the report. The pH of the resulting biochar also varied depending on the feedstock used. The pH values of the biochar produced in the study ranged from 4.6 to 9.3 [79].

Weber et al. reported that temperature variationfrom 200 to 400 °C has great influence on the properties of biochar such as porosity, surface area, etc. [80]. Moreover, as the temperature increases, porosity and particle size of the biochar also increases [81]. The smaller particles have larger surface area and better CEC value [82].The CEC value also depends on the type of raw material used for preparation of biochar.

In addition to the raw material, the decomposition temperature in the production of biochar is also a key factor affecting the adsorption of heavy metals on biochar. It was observed that as the pyrolysis temperature increases, the functional group containing oxygen decreases thereby altering the adsorption efficiency of the biochar. Moreover, the pH of the solution also plays a significant role in deciphering the adsorption capacity of the adsorbent; it can increase or decrease the adsorption capacity based on the model metal impurity. Chen et al. elucidated that the impact of pH on the adsorption of metal ions of copper, zinc, and lead onto biochar derived from corn straw and hardwood. Results obtained revealed that enhancement in the pH from 2.0 to 5.0 enhances the adsorption capacity of metal cations, while at pH above 5.0 the adsorption capacity decreased due to the formation of hydroxide complexes [83]. In contrast, a decrease in pH resulted in an increase in the removal of Cr, as reported by Zhang et al. The reason for this was thought to be the electrostatic interaction of the negative charge of the chromate ion with the positive charge of the biochar at low pH [84].

## 5. Adsorption Mechanism

As indicated above, biochar possesses excellent adsorption capacity towards the remediation of several noxious pollutants whether it is organic or inorganic. Thus, based on the remediation activity it is the need of the hour to document the different adsorption mechanisms under one paper for a better understanding of the readers and researchers across the globe. These adsorption mechanisms not only help us to gain insights but also play a significant role in policy making for enhancing the application of biochar from lab to pilot scale through increasing their removal efficiency and large-scale production. There are certain interactions and forces that act between the target molecule and the surface of biochar.

The interaction between adsorbent and adsorbate majorly depends on the factors such as nature of pollutants, pore nature, pore volume, specific surface area, hydrophobic nature, and surface functionalization, etc.

**Figure 4:**
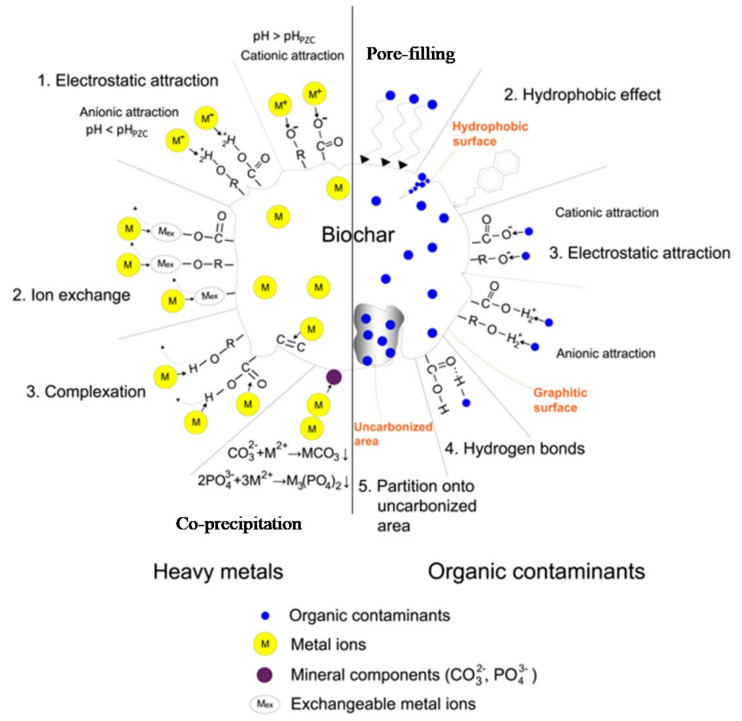
Mechanism of adsorption of various pollutants by biochar (Reprinted with permission from ref. [1], licensed under: CC by 4.0 http://creativecommons.org/licenses/by/4.0/, accessed on 11 January 2023).

The different adsorption mechanism involved during biochar–pollutants interaction was shown in Figure 4. Based on the interaction with different organic and inorganic pollutants, they were classified as coagulation, precipitation, ion exchange, electrostatic interaction, hydrophobic interaction, pore-filling interaction, and hydrogen bond formation.

### 5.1. Complexation

This mechanism involves transition metal to ligand interaction thereby leading to the formation of complex chelate compounds. The oxygen-containing groups at the surface sites interacts with the free orbital of transition metals to form complexes [85]. Further, due to oxidation of biochar surface oxygen content over the time also increase thereby enhancing metal complexation [86]. Liu et al. showed that biochar produced at low temperatures (300 °C) has a high affinity for complexation compared with biochar produced at high temperatures (700–900 °C) [64]. Moreover, from the literary evidences it was observed that this phenomenon is more common in biochar derived from plants rather than animals [87].

### 5.2. Precipitation

This is the most prevalently followed mechanism for adsorptive removal of heavy metals ions onto the biochar. The solid precipitates either in the solution or the adsorbent surface were formed under this mechanism. Further, the heavy metals having ionization potential in the range of 2.5–9.5 eV generally adsorbed on to the biochar surface following the precipitation mechanism [88]. Puga et al. observed that the removal efficiency of biochar following precipitation mechanism majorly depends on the pyrolysis temperature and more research in this is essentially required to optimize this parameter [89]. Moreover, alkaline biochar enhances the precipitation mechanism due to the presence of more active sites that are electronegative in nature thereby enhancing cation adsorptive removal [16,90].

### 5.3. Ion Exchange

This involves the exchange of ions between the solid-liquid interface. The heavy metal ions present in the liquid phase were moved from the liquid phase to the solid phase, i.e., biochar active sites. It is a reversible process between adsorbate and adsorbent [17]. Further, to maintain the electrical neutrality of the aqueous solution, the exchange of ions takes place. Moreover, the removal efficiency of adsorbent in this mechanism majorly depends on two factors, i.e., model pollutant size and type of surface functionalization [87,91].

### 5.4. Electrostatic Interaction

This mechanism is based on the attraction and repulsion of charges, which is the essence of ionic bond formation. If the pollutant to be treated is ionic or readily ionizable, the electrostatic interaction begins to dominate. The cationic pollutants can be easily adsorbed on the negatively charged surface of biochar [19]. The predominance of this mechanism depends on the pH of the solution and the zero charge point (ZCP) of biochar. At pH less than PZC, biochar shows positive charge on its surface promoting the adsorption of negatively charged pollutants. At pH greater than PZC, biochar shows negative charge on its surface favouring the adsorption of negatively charged pollutants. Electrostatic interaction has also been described as an adsorption mechanism for negatively charged Cr (VI) on the positively charged surface of biochar [92].

### 5.5. Hydrophobic Interaction

‘Hydrophobic’ means water-repellent or water-phobic and arises from the presence of nonpolar groups (C-H) in a molecule [93]. This mechanism is used for the adsorption of hydrophobic or neutral organic compounds through hydrophobic interactions. The hydrophobic nature of biochar can be explained by a decrease in oxygen, hydrogen, nitrogen, and sulphur content and an increase in carbon content due to carbonization [94]. The increase in pyrolysis temperature during the production of biochar leads to a decrease in the number of polar groups and enhances the hydrophobic nature of biochar [1]. The adsorption of oil on the surface of biochar occurs mainly due to hydrophobic interactions. Kandanelliet al. [95] adsorbed crude oil on the surface of biochar from rice husk by hydrophobic interaction.

### 5.6. Pore-Filling Interaction

The porous nature of the biochar is the key reason behind the pore-filling interaction mechanism. Biochar possess different types of pores and based on the pore dimensions, the pores that are <2 nm in diameter represent micropores, pores that are in the range 2–50 nm in diameter fall under the category of mesopores, while pores >50 nm in diameter represent macropores [96]. Further, the pore size of the biochar can be controlled by the number of factors which involves synthesis mechanism, pyrolysis temperature, pre- and post-treatment methods and based on the model pollutants size removal efficiency of the adsorbent can be varied during diffusion and adsorption [87]. This mechanism also depends on the polarity of the contaminant [1]. To achieve high adsorption by pore-filling, biochar should have a low volatile content. Ma et al. adsorbed sulphamethoxazole on biochar from sewage sludge. The mechanism of pore-filling was found to be responsible for this adsorption [97].

### 5.7. Hydrogen Bond Formation

Numerous toxic pollutants are adsorbed by the formation of hydrogen bonds on the surface of biochar. The formation of hydrogen bonds occurs due to the negatively charged surface of biochar by the presence of -OH groups [98]. Phenolic compounds, pesticides, dyes, etc., form hydrogen bonds with biochar [99]. The functional groups present either on the surface of biochar or organic pollutant can participate in the formation of hydrogen bond. Groups such as amine (-NH) and hydroxyl (-OH) serve as H-donor. On the other hand, benzene rings, F, N, and O act as H-acceptors for H-bonding to make it more clear in the revised manuscript. The interaction in this process is irreversible because the bond formed is strong [17].

## 6. Applications of Biochar

Rapid industrialization and increased population have led to an increase in concentration of noxious pollutants (organic and inorganic) in the environment. The pollutants can be categorized as organic (e.g., dyes, agrochemicals, etc.) and inorganic (heavy metals).Biochar is a powerful, environmentally friendly, and cost-effective means of combating the current pollution crisis. Due to its versatility, biochar has been widely used to treat various pollutants in recent years. So far, from past few decades biochar is known to be a low-cost material that was used as successful adsorbent for the remediation and abatement of noxious impurities such as organic dyes, heavy metals, oil spillages, pesticides, carbon dioxide, etc. (Table 2). The exceptional properties of biochar such as large surface area, porosity, long-term stability, etc., are the main factors for its use as an adsorbent. The properties of biochar can be adjusted depending on the desired application and the analyte to be treated. The type of contaminant/impurity (polar/nonpolar, cationic/anionic, hydrophobic/hydrophilic, and organic/inorganic) also affects the efficiency and applicability of biochar. The following are some areas where biochar has contributed to environmental remediation as an adsorbent:

### 6.1. Remediation of Organic Pollutant

Exponential growth in textile industries lead to the disposal of organic impurities such as dyes, phenolic compounds, PAHs, PPCBs, chlorophenols, etc., into the nearby aquatic resources. These pollutants possess negative impact on the environment as well as human health. Due to their potential threat to the environment, it is imperative to treat them before they are released into the environment. Biochar has the potential to remove hazardous and poorly degradable organics through an adsorption mechanism. Adsorption of organic pollutants mainly depends on the surface properties of biochar and the type of pollutant. For example, the adsorption of dyes on biochar strongly depends on whether the dye is cationic or anionic.

#### 6.1.1. Remediation of Dyes

Reports indicate that the pH of the solution has a significant effect on the adsorption capacity of biochar depending on the type of dye. It was found that the pH < 2 (acidic) is favourable for the adsorption of anionic dye, because the increase in H^+^ ions leads to a positive charge on the biochar surface. This promotes electrostatic attraction between the anionic dye and the positively charged biochar surface. For cationic dyes, the pH > 2 is preferred because at this pH the presence of OH^−^ ions increases. As a result, the surface of biochar becomes negatively charged. As a result, enhancement of adsorption of positively charged cationic dyes and negatively charged biochar is observed due to electrostatic interaction [100,101,102]. The biochar synthesized from litchi peel was used as adsorbent for the remediation of anionic and cationic dyes, i.e., for Congo red and malachite green from the aqueous solution, the results obtained revealed the great removal efficiency of ~404.4 and 2468 mg/g, respectively [47]. Mian et al. demonstrated the removal of dyes from biochar prepared from sewage sludge. They pre-treated and post-treated the starting material and the obtained biochar by chemical treatment. They found that the biochar served as an excellent adsorbent for the removal of Acid Orange, Rhodamine B, Methylene Blue, and Methyl Orange [103].

**Table 2:** Sources, time, adsorbent dosage, targeted pollutant molecules, and adsorption efficiency of biochar.

Type of Pollutant		Source of Biochar	Time	Adsorbent Dosage (g/L)	Targeted Molecule	Efficiency	Ref.
Dyes	Cationic	Cattle manure	24 h	1.25	Methylene blue	241.9 mg/g	[104]
		Municipal waste	360 min	5	Methylene blue	7.2 mg/g	[105]
		Rice husk	2 h	0.2	Malachite green	99.98%	[106]
		Cactus	210 min	0.6	Malachite green	1341 mg/g	[107]
		Date palm	24 h	1	Crystal violet	27.4 mg/g	[108]
		Rice straw	15 min	0.001	Methylene blue	94.45%	[109]
			20 min		Crystal violet	92.07%	
		Animal waste	4 h	2.5	Basic red 9	52.3 mg/g	[110]
		Litchi peel	12 h	1	Malachite green	2468 mg/g	[47]
	Anionic				Congo red	404.4 mg/g	
		Orange peel	24 h	3.0	Congo red	93%	[111]
		Pine nutshell	600 min	0.4	Acid chrome blue	27.24 mg/g	[112]
Oil		Popped rice	30 min	-	Kerosene	6.51 g/g	[113]
		Goat hair	120 min	1.5	Diesel	466 mg/L	[114]
					Crude oil	510 mg/L	
					Kerosene	367 mg/L	
					Petrol	344, mg/L	
		Tyres	-	-	Crude oil	8.89 g/g	[115]
		Mango shell	75 min	50	Crude oil	95%	[116]
		Crab shell	240 min	0.2	Diesel oil	93.9 mg/g	[42]
		Textile sludge	-	-	Cooking oil	120.1 mg/g	[117]
		Coconut coir	100 min	12	Crude oil	99.9%	[118]
		Water hyacinth	60 min	1	Fuel oil	80%	[119]
		Commercially available	60 min	10	Crude oil	11 g/g	[120]
Other organic molecules		Wood chips			Naphthalene	76%	[121]
		Astragalusmongholicus	12 h	2	Ciprofloxacin	40.11 mg/g	[122]
		Bull manure	180 days	-	Lincomycin	99%	[123]
		Pine wood	48 h	0.5	Tetracycline	163 mg/g	[124]
		Pine chips	7 days	0.05	Ibuprofen	20 µM	[125]
		Pine saw dust	4 h	5	p-nitrophenol	99.61%	[126]
		Paper sludge	143 min	4	2,4- dichlorophenol	99.95%	[127]
		Rice straw	120 min	1.2	Tetracycline	98.33 mg/g	[128]
		Cotton gin waste	1500 min	5	Sulphapyridine	70%mg/g	[129]
					Docusate	98%mg/g	
					Erythromycin	74%mg/g	
		Orange peel	2 days	6.25	p-nitrotoluene	110 mg/g	[130]
		Sludge	120 min	0.1	Sulfamethoxazole	5.43 × 10^−3^ µg/g	[97]
		Enteromorphaprolifera	24 h	0.1	Pyrene	93.5%	[131]
		Oak	14 days	2	Catechol	59%	[132]
		Wood	30 min	-	Butylbenzyl phthalate	10^5^ µg/g	[133]
		Soybean stover	48 h	0.3	Trichloro ethylene	25.38 mg/g	[134]
		Rape stalk	48 h	0.05	Tetracycline	35.90 mg/g	[135]
		Hair waste	30 min	-	Amoxicillin	90%	[136]
					Diclofenac	80%	
Heavy metals		Corn cob	24 h	2	Nitrate	32.33 mg/g	[137]
		Sewage sludge	-	10	Cr	3.0 mg/g	[138]
		Pine bark	5 h	-	Pb	4.25 mg/g	[139]
		Oak wood	-	-	Pb	75.8 %	[140]
		Cotton stack	-	-	Cd	77.66%	[141]
		Bamboo	12 days	-	Cd	79.6%	[142]
		Yak manure	48 h	2	Pb	76.41 mg/g	[143]
		Plantain peel	150 min	10	Pb	4 mg/g	[144]
		Fish scale	24 h	2	Cu	39.39 mg/g	[44]
		Hard wood	24 h	5	Cu	6.79 mg/g	[83]
					Zn	12.52 mg/g	
		Dairy manure	24 h	1.5	Pb	175.53 mg/g	[145]
		Sugarcane straw	24 h	4	Cd	16 mg/g	[146]
					Zn	6 mg/g	
		Reed	2 h	16	Cd	86.8%	[147]
					Pb	83.5%	
		Walnut shell	120 min	3	Ni	13.25 mg/g	[148]
		Rice husk	24 h	-	As (III)	85%	[149]

#### 6.1.2. Remediation of Oil

Oil spills have dangerous effects on the marine ecosystem and on the land (soil). The oil adsorption capacity of biochar can be mainly related to its hydrophobic/oleophilic nature, which can be estimated by measuring the water contact angle [150]. In addition to hydrophobicity, other factors such as porosity, surface area, etc., also play an important role in determining the oil adsorption capacity of biochar. Biochar with high porosity has higher oil removal capacity because the oil is trapped between the porous arrays [95,151]. Biomass with a high lignin content results in biochar that is more hydrophobic and amorphous. This type of biochar shows better oil removal capacity [50]. Gurav et al. prepared pine biochar modified with coconut oil and used it for adsorption of petroleum impurities [152]. They found that the biochar prepared at 700 °C was best for oil adsorption due to its hydrophobic character at higher temperature.

#### 6.1.3. Remediation of Other Organic Contaminants

Organic molecules such as benzene, penicillin, chlorofluorocarbons, etc., when released into the environment, have adverse effects on the environment and human health. Adsorption of organic molecules with biochar is one of the most effective methods to treat toxic molecules. 2,4-dichlorophenol was adsorbed from water using wheat husk-derived biochar by Kalderis et al. [127]. In addition to natural biochar, EB is also widely used for the removal of toxic organic molecules [153]. Dai et al. performed adsorption of tetracycline from water using magnetic biochar made from rice straw. Biochar treated with cerium trichloride was used to treat levofloxacin (drug). Cerium treatment increased the oxygen-containing functional group on the biochar surface, resulting in improved adsorption performance from 37.80 mg/g to 98.33 mg/g [128].

### 6.2. Remediation of Inorganic Pollutants

Inorganic pollutants mainly include heavy metals such as lead (Pb), mercury (Hg), cadmium (Cd), zinc (Zn), etc., which enter the environment through industrial wastewater [141]. The presence of these metals, even in very small amounts, can have a significant impact on the environment. Adsorption has proven to be a universal, fast, convenient and effective method for the removal of these hazardous pollutants [142].

Biochar has a strong affinity for heavy metals due to its surface area, porosity, and the oxygen-containing functional group on its surface. The mechanisms involved in the immobilization of heavy metals are generally ion exchange, precipitation, electrostatic interaction, and complexation. The adsorption performance depends on the type of heavy metal (cationic/anionic) and the amount of oxygen-containing functional groups (carboxylate and hydroxyl) on the surface of biochar. The size of the pores and the surface area of biochar affect the performance of biochar [154]. The feedstock used as raw material for biochar production also affects the heavy metal sorption capacity of biochar. Biochar produced from agricultural and forestry residues improve the sorption capacity for heavy metals. This may be attributed to the numerous oxygen-containing functional groups on the surface of biochar, which provide negatively charged surface sites favourable for adsorption of heavy metals [58]. Mohan et al. reported that biochar prepared from oak bark increased the adsorption sites for Pb^2+^, resulting in its better adsorption on the surface of the biochar [155]. The use of animal waste such as poultry litter for biochar production increases the ash and inorganic content of the biochar. This improves the binding of heavy metals to the surface of the biochar. Mineral components such as CO_3_^2−^ and PO_4_^3−^ from the feedstock serve as additional adsorption sites for the adsorption of heavy metals on biochar [53]. The H_2_O_2_-modified biochar from yak manure was used by Liu et al. as an adsorbent for the removal of heavy metals. They concluded that the modified biochar provided excellent adsorption for the removal of heavy metals such as Pb^2+^, Cu^2+^, Cd^2+^, and Zn^2+^ [143].

## 7. Engineered Biochar (EB)

**Figure 5:**
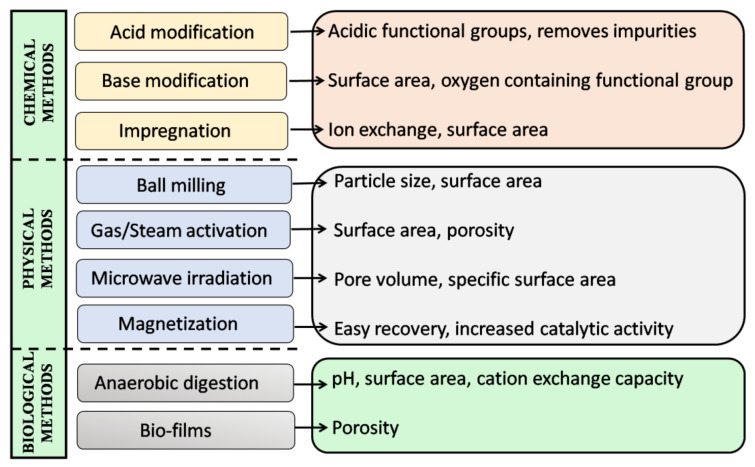
Schematic representation of different methods used for preparation of engineered biochar and their effect on the properties of biochar.

The main advantage of biochar is that its properties can be adjusted depending on the substance to be treated. The term “engineered biochar” is used for biochar that has been modified or activated using various techniques. In recent years, there has been an increase in the development of EB to expand the applications of biochar. Modification techniques for biochar production can be divided into chemical, physical, and biological. Techniques such as acid/alkali treatment, impregnation of the surface with other materials, ball milling, acetylation, etc., are used to tune the properties of biochar [156,157]. These modification techniques can be used before (biomass treatment/specific reaction temperature) or/and after (treatment of pure biochar) the production of biochar [157].An overview of all the methods and their effect on the properties of biochar is provided in Figure 5.

Qui et al. demonstrated the improvement of biochar by treatment with NaOH or HCl [158]. Similarly, Ahamad et al. and Nazifa et al. reported that engineered biochar (EB) is produced by adding iron and zinc. Treating the biochar with acid, bases, or metals affects the properties of biochar. It improves the formation of oxygen-containing functional groups, surface area, pore volume, and surface charge. This in turn increases the adsorption efficiency of the biochar [159,160]. Modification of surface charge by impregnation with Fe salt is another strategy to improve the performance of EB [161]. Thus, one can modify and improve the properties of biochar by the above chemical treatments and monitor the parameters that affect the properties of biochar, and thus develop a suitable adsorbent with the desired properties for adsorption of a range of pollutant molecules.

Physical methods for producing EB include processes such as ball milling, gas/steam activation, and magnetization. Ball milling is a technique that grinds biochar into fine nanoscale particles [162]. This technique is widely used to increase the surface area, porosity, and acidic functional groups on the surface of biochar. The modified biochar obtained by this method exhibits increased adsorption capacity due to the increased surface area and porosity [17,153]. The electrostatic interaction and surface complexation with the pollutants are favoured by the increase in acidic functional group on the surface of biochar [162]. Gas/steam activation produces H_2_ and CO_2_ by surface oxidation, which is used to activate the surface of biochar. This biochar engineering technique is used to induce porosity formation, increase surface reactivity, increase specific surface area, and remove residues trapped due to incomplete combustion during pyrolysis [163]. Biochar magnetization involves the introduction of transition metals (Fe, Co, Ni, etc.) and their oxides into the biochar matrix [164]. This is an effective strategy to solve the problem of separating powdered biochar from the environmental medium. Due to this additional advantage, magnetic biochar has been shown to be very effective in removing heavy metals and organic pollutants from an aqueous medium (wastewater) [165,166]. The production of magnetic biochar requires expertise, as improper handling can lead to clogging of the pores and a reduction in surface area [167].

EB can be produced by various chemical processes such as oxidation and synthesis of biochar-based composites. Oxidation with acids (HCl, H_2_SO_4_, and H_2_O_2_), bases (NaOH and KOH) or other oxidants (KMnO_4_ and Fe(III)) is used to charge the surface properties of biochar [168]. Acid/base modification increases the availability of oxygen- and carbon-containing functional groups, optimizes surface electrostatic attraction, surface precipitation, and surface complexation [169]. When oxidants are used to activate biochar, the surface area and pore size distribution improve. Another technique to obtain EB is the synthesis of biochar-based composites with metal oxides, chitosan, amino groups, etc. [167]. The composites are prepared to improve the physicochemical properties of biochar and increase its efficiency in a particular application. For example, the composites of biochar and metal oxides have good electrostatic ability, good ion exchange, and good precipitation capacity, so they provide biochar better adsorption properties [170].

Biological methods for EB synthesis mainly involve the use of microorganisms to obtain biochar with desired properties. The microorganism can form a biofilm on the inner and outer surfaces of biochar [167]. This affects the pore distribution of biochar and improves its adsorption and degradation capacity for various organic pollutants [171]. One study reported that biochar with active biofilm is a promising adsorbent for the removal of pharmaceuticals from wastewater systems [172]. Studies have also shown that biochar modified by biological methods can be effectively used as a biofiltration medium in wastewater treatment [173].

## 8. Challenges and Future Research Direction with Regard to the Sustainable Development Goals (SDGs)

Although much research has been performed on biochar, there are some issues and challenges that need to be addressed. The economical and technical aspects of biochar need further study before it can be used on a large scale. The efficiency and mechanism of biochar as an adsorbent have been extensively studied, but more attention should be paid to the desorption of pollutants. Regeneration of biochar is another area that can be further explored. Studies on biochar are mostly conducted in the laboratory and in the presence of only one pollutant. Therefore, adsorption by biochar in the presence of coexisting analyte should be conducted and studied in the future.

In addition, achieving clean water and sanitation is one of the United Nations’ 17 Sustainable Development Goals (SDGs). To take a step towards the Sustainable Development Goal (SDG 6: Clean Water and Sanitation), the use of biochar as an adsorbent for wastewater treatment in this review was discussed. Biochar is reportedly a sustainable solution to the prevalent problem of water pollution. Biochar is derived from biomass/waste and has been shown to be a potential adsorbent for heavy metals, dyes, organic molecules, oil, etc.

## 9. Conclusions and Recommendations

The present review dealt with the prospects of biochar reducing various organic and inorganic pollutants through adsorption. In recent years, biochar has received alot attention for its application as an excellent adsorbent for wastewater treatment and removal of toxic dyes, heavy metals, agrochemicals, pharmaceuticals, etc. Potential sources of biochar as well as the synthetic methods such as pyrolysis, HTC, torrefaction, carbonization, used to produce biochar have been discussed in detail. Various characterization techniques used to analyse the physical and chemical properties of biochar show that biochar is an excellent adsorbent owing to its properties such as surface area, porosity, surface functional groups, etc. A detailed study of the adsorption mechanism shows that the analyte interacts with biochar through hydrogen bonding, electrostatic interaction, ion exchange, hydrophobic interaction, and pore-filling, which mainly depends on the nature of the analyte. Studies on EB show that the adsorption properties of biochar can be modified depending on the analyte to be treated. Biochar has proven to be a green adsorbent as it is derived from bio-waste, and offers researchers a wide scope for research work.

## Data Availability

Not applicable.
